# Past and present dynamics of sorghum and pearl millet diversity in Mount Kenya region

**DOI:** 10.1111/eva.12405

**Published:** 2016-09-23

**Authors:** Vanesse Labeyrie, Monique Deu, Yann Dussert, Bernard Rono, Françoise Lamy, Charles Marangu, Dan Kiambi, Caroline Calatayud, Geo Coppens d'Eeckenbrugge, Thierry Robert, Christian Leclerc

**Affiliations:** ^1^ UMR AGAPCIRADMontpellierFrance; ^2^ Ecologie, Systématique et EvolutionUMR 8079 CNRSUniversité Paris‐SudOrsayFrance; ^3^ KALROEmbuKenya; ^4^ Department of BiologieUVSQVersaillesFrance; ^5^ ICRISATNairobiKenya; ^6^ Sorbonne Universités, UPMC Univ Paris06, IFDParis Cedex 05France; ^7^ Present address: UPR GREENCIRAD34398MontpellierFrance; ^8^ Present address: UMR 1065 Santé et Agroécologie du VignobleINRA33140Villenave d'OrnonFrance; ^9^ Present address: CIMMYT00621NairobiKenya; ^10^ Present address: ABCICP.O. Box100882‐00101NairobiKenya

**Keywords:** crop diversity, farmers’ seed systems, gene flow, population genetics, social organization

## Abstract

Crop populations in smallholder farming systems are shaped by the interaction of biological, ecological, and social processes, occurring on different spatiotemporal scales. Understanding these dynamics is fundamental for the conservation of crop genetic resources. In this study, we investigated the processes involved in sorghum and pearl millet diversity dynamics on Mount Kenya. Surveys were conducted in ten sites distributed along two elevation transects and occupied by six ethnolinguistic groups. Varieties of both species grown in each site were inventoried and characterized using SSR markers. Genetic diversity was analyzed using both individual‐ and population‐based approaches. Surveys of seed lot sources allowed characterizing seed‐mediated gene flow. Past sorghum diffusion dynamics were explored by comparing Mount Kenya sorghum diversity with that of the African continent. The absence of structure in pearl millet genetic diversity indicated common ancestry and/or important pollen‐ and seed‐mediated gene flow. On the contrary, sorghum varietal and genetic diversity showed geographic patterns, pointing to different ancestry of varieties, limited pollen‐mediated gene flow, and geographic patterns in seed‐mediated gene flow. Social and ecological processes involved in shaping seed‐mediated gene flow are further discussed.

## 1 Introduction

Most of the world crop diversity continues to be maintained in smallholder farming systems, which represent 80% of arable lands in southern countries (Altieri, 1999). Increasing our knowledge on crop diversity dynamics in these systems is crucial for the conservation of crop genetic resources. These dynamics are driven by the interaction among biological, ecological, and human‐driven processes, occurring on different spatiotemporal scales (Brush, 2000) and shaping selection forces and gene flow (Leclerc & Coppens d'Eeckenbrugge, 2012; Mercer, Martínez‐Vásquez, & Perales, 2008; Orozco‐Ramírez, Ross‐Ibarra, Santacruz‐Varela, & Brush, 2016; Perales, Benz, & Brush, 2005).

Among biological drivers, crop reproductive biology has a major impact on the local and short‐term dynamics of genetic diversity. Pollen‐mediated gene flow may lead to genetic homogenization within and among fields, particularly for allogamous crops such as pearl millet (*Pennisetum glaucum* (L.) R. Br.; Busso et al., 2000; Robert et al., 2003; vom Brocke, Christinck, Weltzien, Presterl, & Geiger, 2003). On the contrary, for the mostly self‐pollinating sorghum (*Sorghum bicolor* (L.) Moench), restricted cross‐pollination among varieties preserves their genetic distinctiveness on a local scale (Barnaud, Deu, Garine, McKey, & Joly, 2007), although admixture among varieties may occur (Deu et al., 2014).

Among human factors, seed exchanges and sales are major drivers of gene flow within and among villages (Badstue et al., 2007; Pressoir & Berthaud, 2004a; Thomas, Dawson, Goldringer, & Bonneuil, 2011; vom Brocke et al., 2003), and sometimes over long distances (Coomes et al., 2015). These seed dissemination networks are strongly dependent on social organization. Previous studies showed the importance of kinship in northern Cameroon (Wencélius & Garine, 2014), neighborhood in Ethiopia (McGuire, 2008), and ethnicity in the Mount Kenya region (Labeyrie, Thomas, Muthamia, & Leclerc, 2016). These “informal” seed systems, as opposed to the “formal” ones, either institutional or commercial (Almekinders, Louwaars, & De Bruijn, 1994), present a limited geographic extent (Hodgkin et al., 2007). Consequently, they are often circumscribed to relatively uniform agroecological areas (Bellon, Hodson, & Hellin, 2011; Samberg, Fishman, & Allendorf, 2013).

Furthermore, crop adaptation to agricultural practices and local conditions, through farmers’ seed selection, is also involved in crop diversity patterns (Mercer et al., 2008; Pressoir & Berthaud, 2004b). The combination of seed selection and diffusion processes was notably involved in diversity patterns of barley in Ethiopia and rice in Yunnan, where particular landraces were circumscribed to specific elevations (Samberg et al., 2013; Xiong et al., 2010). Similar results were also reported for maize in Mexico where genetic differentiation among elevation zones was observed at a regional scale (van Heerwaarden, 2007), while other studies found that ethnolinguistic differences have a stronger impact than elevation at a local scale (Orozco‐Ramírez et al., 2016). For sorghum, most studies reported a very weak relationship between neutral genetic diversity patterns and elevation or agro‐climatic zones at a country scale (Ayana, Bryngelsson, & Bekele, 2001; Deu et al., 2008; Mutegi et al., 2011). For pearl millet, this topic is still understudied, but recent studies found a relationship between allelic frequencies at a gene associated with flowering time and annual rainfall variations in Niger (Mariac et al., 2011).

Lastly, on a wider spatiotemporal scale, past human migrations have also largely contributed to present‐day diversity patterns, as was remarkably highlighted for sorghum across Africa (Westengen, Okongo, et al., 2014), for the diffusion of banana from New‐Guinea to West Africa (Perrier et al., 2011), of maize in the Americas (Vigouroux et al., 2008), and of sweet potato from South America to Polynesia (Roullier, Benoit, McKey, & Lebot, 2013).

The interactions among these multiple biological, ecological, and social processes make crop populations especially interesting models for evolutionary genetic studies. The complexity that characterizes crop diversity dynamics calls for integrative interdisciplinary studies, at the crossroad of population genetics, ecology, and anthropology (Lansing et al., 2007). However, most studies implemented on a large spatial scale have focused on past processes, such as crop diffusion through human migrations, while those implemented on a local scale have focused on current practices, mainly seed exchanges or selection. Past and current processes have rarely been analyzed together in the same study to understand local diversity dynamics.

Landscape approaches (Manel, Schwartz, Luikart, & Taberlet, 2003) have proved to be integrative tools, adapted to disentangle the effects of the various processes involved in crop diversity dynamics (Samberg et al., 2013). They were implemented in the present study to investigate diversity patterns in sorghum and pearl millet on the eastern side of Mount Kenya. These two central crops of Mount Kenya agroecosystems are managed similarly by farmers. They strongly differ in their breeding systems, which allowed assessing the effect of pollen‐mediated gene flow on crop diversity dynamics. Besides, the Mount Kenya region offers an interesting setting to study the effect of social and ecological processes on seed‐mediated gene flow. On the one hand, elevation gradients along the slope of Mount Kenya (Jaetzold, Schmidt, Hornetz, & Shisanya, 2007) may shape seed dissemination networks, as ecological adaptation is potentially involved in farmers’ varietal choice (Bellon et al., 2011; Samberg et al., 2013). On the other hand, limited seed exchanges are expected among the different ethnolinguistic groups living along a south–north gradient in the area (Middleton & Kershaw, 1965). Indeed, a previous study on a contact zone among three groups in the area showed that seed diffusion networks were largely channeled by ethnolinguistic organization (Labeyrie et al., 2016). Our field setting thus offered the possibility to implement the double comparative approach recommended by Leclerc and Coppens d'Eeckenbrugge (2012) to assess the effect of agroecological conditions and ethnolinguistic organization on sorghum and pearl millet genetic diversity dynamics. Furthermore, the Mount Kenya region also allows exploring the effect of past diffusion processes on sorghum diversity patterns as several introductions probably accompanied the migrations of different farming populations around the 18th century (Middleton & Kershaw, 1965; Mwaniki, 1973).

Observed patterns of crop diversity can result from different processes (Sagnard et al., 2008). This study aimed at highlighting this complex interplay between ecological and human‐driven process involved in crop diversity dynamics, based on the joint analysis of in‐depth farm surveys and genetic analyses at both local and continental scales. First, patterns of sorghum and pearl millet diversity were compared at the Mount Kenya scale. An analysis of both named varieties and neutral genetic diversity patterns, combined with that of local seed systems, enabled us to discuss the processes involved in local diversity dynamics. Secondly, we focus on the sorghum model to investigate past diffusion dynamics by comparing Mount Kenya genetic diversity to that of the African continent. Finally, we discuss the respective effects of the various past and present processes involved in diversity dynamics.

## 2 Materials and Methods

### 2.1 Ethic statement

This was a collaborative study with the Kenya Agricultural and Livestock Research Organization (KALRO), which has the national mandate for collecting and conserving all plant genetic resources and documenting all accompanying information. Based on this mandate and given that KALRO was a partner of the present study, no specific permission was required to undertake it. Although KALRO has not designated any ethical review board, it has equivalent committees and administrative organs that review proposed research activities before granting approval. Local government administrative as well as agricultural extension officers were informed of the study and kept updated on its activities. During the survey, the mandate given to KALRO as well as the importance of the study, both nationally and globally, was explained to farmers. According to KALRO's procedures, prior informed consent was obtained verbally and not recorded. Farmers were told that the process would only involve anonymous information.

### 2.2 Site of study

**Figure 1:**
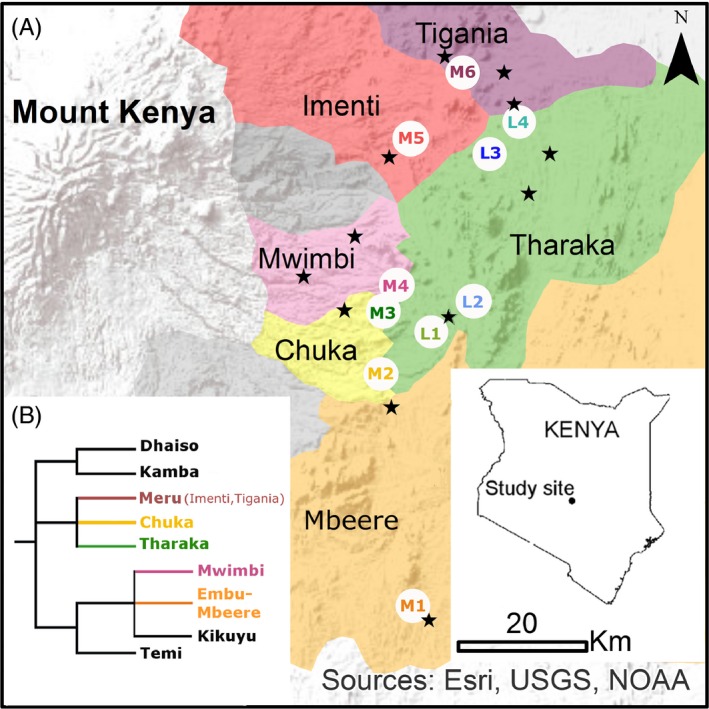
Location of the study area and ethnolinguistic diversity. (a) Location of the study sites (lowland sites: L1–L4, midland sites: M1–M6) and major local markets (symbolized by stars). (b) Linguistic classification of the central Kenya Bantu languages (Hammarström et al., 2015)

This study was conducted on the eastern slope of Mount Kenya, in ten sites located in the Meru, Tharaka‐Nithi, and Embu counties (Fig. 1), and along two altitudinal transects. Four sites (L1–L4) were selected at elevations around 750 m (hereafter referred as lowlands), in the area mainly inhabited by the Tharaka ethnolinguistic group. The southernmost and northernmost sites were distant of 35 km on this low‐altitude transect. At ca. 950 m (hereafter referred as midlands), six sites (M1–M6) were selected, each one being located in a distinct ethnolinguistic group: Mbeere, Chuka, Tharaka, Mwimbi, Imenti, Tigania, from south to north. The southernmost and northernmost sites of this upper transect were distant of 84 km.

Two large roads cross the lowlands at around 750 masl. and the highlands at around 1,500 masl. Midlands have limited road access, being served only by dirt roads on ridgetops. Major Chuka and Meru towns are located in the highlands, where economic activity is mainly based on cash crops (tea, coffee, and *khat*). In midlands and lowlands, rural populations base their livelihood on subsistence economy, relying on small‐scale and low‐input traditional crop–livestock farming systems (Jaetzold et al., 2007). Farmers sell and buy seed on the local markets, which are mostly located at less than 10 km from their home (Fig. 1). Indeed, farmers mainly walk to the market as transport is costly and difficult in the mountainous midlands.

The study area presents bimodal rainfalls, with the long rains from March to May and the short rains from October to December, and cropping seasons correspond with these periods. Sites M1, M2, L1, and L2 are located in the area defined as “lower‐midlands livestock and millet” zone, presenting a semiarid climate, the M5 site in the “lower‐midlands cotton” zone, with a semi‐humid climate, while sites M3, M4, M6, L3, and L4 are located in the “lower‐Midlands marginal cotton zone,” with an intermediate climate (Jaetzold et al., 2007).

In our study area, farmers manage low‐input cropping systems involving pearl millet and sorghum, frequently intercropped with other species, especially grain crops and legumes such as maize (*Zea mays* L.), beans (*Phaseolus vulgaris* L.), cowpeas (*Vigna unguiculata* (L.) Walp.), or mung bean (*Vigna radiata* (L.) R. Wilczek) depending on the agroecological zone. Different varieties of these crops are usually grown within the same plot. Pearl millet and single‐season sorghum varieties can be grown during both cropping seasons (either from October to January or from March to June), but farmers also grow ratoon sorghum varieties that are sown in October, cut down in January to stimulate regrowth, and finally harvested in July. Ratoon sorghum is frequently grown around field borders or terraces (personal observation). According to farmers, they improve soil conservation and stability, increase soil water availability, and enable them to save time and labor (Wilson, 2011).

The outstanding ethnolinguistic diversity on the eastern slope of Mount Kenya makes it an ideal place for the study of the relationship between crop and human societies. In this area, all farmers speak languages belonging to the Central Kenya Bantu language cluster (Hammarström, Forkel, Haspelmath, & Bank, 2015). However, they present major historical, cultural, and linguistic differences (Heine & Möhlig, 1980; Lambert, 1947; Middleton & Kershaw, 1965). The original Meru linguistic and cultural cluster is constituted of five ethnolinguistic groups, referred as “tribes” by colonial observers, among which are the Imenti (site M5) and Tigania (site M6) groups (Hammarström et al., 2015). The Meru probably settled there around the 18th century (Middleton & Kershaw, 1965). The Mwimbi group (M4 site) is linguistically and culturally distinct but closely related to the Meru, as well as the Tharaka (sites M3 and L1–L4) and Chuka (site M2) who are allied and consider that they descend from a common ancestor (Fadiman, 1993). The Mbeere (site M1) are part of the Embu cluster and have a different history from that of the Meru according to oral reports (Mwaniki, 1973).

### 2.3 Inventories and sampling

#### 2.3.1 Farm survey

Inventories of sorghum and pearl millet named varieties were conducted in December 2006 and January 2007, before harvest. A total of 189 farms were surveyed in the ten sites, with a mean of 19 farms (min = 15, max = 21) taken at random in each site. Among farmers, 42.3% were men. The large majority of interviewed farmers were from the dominant ethnolinguistic group in the area, and husbands and wives belonged to the same group.

During the interviews, the local names of all sorghum and pearl millet varieties sown in October 2006 were recorded. For each named variety, we asked farmers whether it was local (landrace) or exogenous (exotic origin or formal crop improvement system). For sorghum, we also asked whether it was a single‐season or a ratoon variety. Harmonization of variety names was later performed by a native Central Kenya Bantu languages speaker to limit errors due to spelling, transcription, and pronunciation differences among languages. A “named variety” hence corresponds to a unit identified using the same name by farmers, within or among sites.

Farmers were also asked whether the seed lot of each variety sown in October 2006 was of self‐produced origin, provided by a relative, a friend, or a neighbor, bought at the market, or obtained from another exogenous origin (i.e., distributed by the government extension service, by NGOs, by local administrators, or through churches). We also collected for each variety the source from which farmers got the variety for the first time (initial source of seeds).

#### 2.3.2 Variety sampling

**Figure 2:**
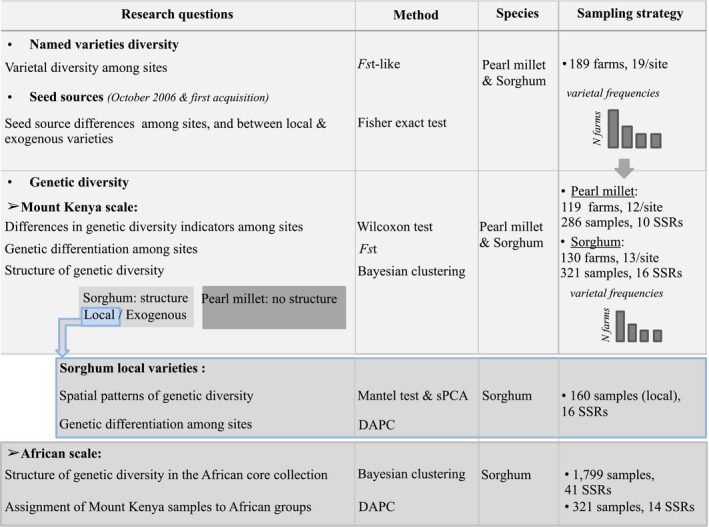
Summary of the sampling strategy and analyses

The strategy for on‐farm germplasm collection was based on varietal frequency at each site of study. For each site, a farm subsample (5–14 farms for pearl millet; 9–16 farms for sorghum) was defined to represent the diversity and frequencies of each variety (Fig. 2; See Tables S1 and S2 for more details). In total, over the ten sites, 286 pearl millet samples, from 15 named varieties, were collected in 119 farms, whereas 321 sorghum samples, from 22 named varieties, were collected in 130 farms. For each named variety grown in each farm, we collected seeds from one to three plants for pearl millet, and from one or two plants for sorghum. Seed samples were collected in January (single‐season sorghum and pearl millet) and July 2007 (sorghum ratoon varieties).

### 2.4 Genetic characterization

Sorghum and pearl millet seeds were grown in the Biosciences Eastern and Central Africa hub located within the International Livestock Research Institute, Nairobi, at room temperature for 2 weeks in plastic potting trays. The ICRISAT team extracted genomic DNA from fresh leaves harvested on a single seedling per sample (321 samples for sorghum and 286 for pearl millet) using a modified CTAB protocol (Mace, Buhariwalla, Buhariwalla, & Crouch, 2003). Quality and quantity were assessed for all DNA samples before shipping them to France.

For sorghum, 16 SSRs were selected for their high polymorphism as revealed in previous studies (Deu et al., 2008; Table S3). Among them, 14 were part of a set of reference microsatellite markers recommended for diversity studies (Billot et al., 2012), and had been used in a worldwide characterization of sorghum genetic diversity (Billot et al., 2013). Their loci were distributed over eight chromosomes of ten. PCR conditions and genotyping on Li‐Cor automated sequencers were as described by Barnaud et al. (2007). Saga GT v. 2.2 (Li‐Cor) was used to determine allele sizes. Three control panel DNA samples were used as standard checks in every PCR and electrophoresis run to facilitate accurate allele calling (Billot et al., 2012). Genotyping was performed at the Montpellier Languedoc‐Roussillon Genopole platform located on the CIRAD campus.

For pearl millet, 12 SSRs that are fairly well distributed on its genetic map were selected for this study (Qi et al., 2004; Senthilvel et al., 2008, Table S3). A standard simplex PCR protocol was used. The final PCR mixture (12.5 μl) consisted of 30 ng of genomic DNA and 10 μl of PCR mix. The PCR mix consisted of 1 μl of 10× *Taq* polymerase buffer (including MgCl2 15 mmol L^−1^), 0.1 μl of forward primer labeled with a fluorochrome (10 μmol L^−1^), 0.15 μl of unlabeled forward primer (10 μmol L^−1^), 0.25 μl of unlabeled reverse primer (10 μmol L^−1^), 0.5 μl of each dNTP (2.5 mmol L^−1^ each), and 0.1 μl of *Taq* polymerase 5 U μl^−1^ (MP Biomedicals); and H_2_O qsp 10 μl. Amplifications were performed in 96‐well thermocyclers using the following program: 5 min at 95°C for initial DNA denaturation followed by 35 cycles of 30 s denaturation at 94°C, 90 s for primers annealing (55–58°C), 30‐s extension at 72°C, and a final extension of 10 min at 72°C. PCR products were pooled by groups of 4 loci and loaded on a capillary sequencer (ABI 3100xl Genetic Analyser). Alleles were scored with GeneMapper v4.0 (Applied Biosystems). Genotypes were manually checked for every individual. Marker data for two highly polymorphic SSR markers (PSMP 2214 and PSMP 2227) were excluded from the final analysis due to scoring ambiguities.

### 2.5 Data analysis

Our analyses are summarized in Fig. 2. First, diversity patterns of both pearl millet and sorghum were characterized based on named varieties on the one hand, and on SSRs markers on the other hand. Seed sources were also investigated for both species. Then, further analyses were conducted to investigate the factors involved in sorghum genetic patterns, at two levels: (i) geographic patterns of sorghum local varieties, avoiding potential confusions related to introductions of exogenous materials, and (ii) relationships between Mount Kenya genetic groups and major African clusters.

#### 2.5.1 Farm survey

To quantify the differences of varietal composition among sites, we computed *F*_ST_‐like estimates based on variety names (Sahri et al., 2014), using the method of Weir and Cockerham (1984) implemented in FSTAT 2.9.3.2 software (Goudet, 2002). The significance of the differences among sites was assessed using a permutation test (3,000 permutations) with a Bonferroni correction. The *F*_ST_‐like estimates were computed based on variety frequencies. In the data table, the presence of a variety in a farm was coded like the presence of a homozygous genotype for a single locus.

The relative importance of seed sources was computed for the seed lots sown in October 2006 at each site (sorghum seed lots: *N* = 313; pearl millet: *N* = 210), as well as for remembered initial seed sources (sorghum: *N* = 169; pearl millet: *N* = 103). We also computed these fractions separately for seed lots identified as local varieties by farmers (October 2006 lots: sorghum: *N* = 165, pearl millet: *N* = 65; initial lots: sorghum: *N* = 86, pearl millet: *N* = 36). Global and pairwise Fisher's exact tests were conducted to compare seed source proportions (October 2006 and initial ones) between local and exogenous varieties, and among sites.

#### 2.5.2 Organization of sorghum and pearl millet genetic diversity on Mount Kenya

The genetic diversity of sorghum and pearl millet samples at each site (one site being considered as a population for each species) was assessed by computing the observed number of alleles and heterozygosity, allelic richness, corrected for sample size, and unbiased gene diversity (expected heterozygosity). These indices were compared among sites using paired pairwise Wilcoxon tests with a false discovery rate correction (R package *stats*). The overall and pairwise genetic differentiations among the ten sites were measured for sorghum and pearl millet using the global fixation index (*F*_ST_, Weir & Cockerham, 1984). The significance of the differences was assessed using a permutation test (3,000 permutations) and corrected using a Bonferroni procedure. Calculations were carried out using FSTAT 2.9.3.2 software.

The organization of genetic diversity was explored for both species, using the Bayesian clustering algorithm implemented in the STRUCTURE 2.3.2 software (Pritchard, Stephens, & Donnelly, 2000). We used the admixture model with correlated allele frequencies, assuming that the genome of each individual resulted from the mixture of *K* ancestral populations. The proportions of each individual genotype originating from each of the *K* ancestral populations (*q*) was estimated for *K* ranging from 1 to 10, with 20 runs for each *K* value. The burn‐in period was set at 500,000 and 1,000,000 iterations were performed. Outputs were summarized using Structure Harvester (Earl & vonHoldt, 2012). To determine the most likely number of ancestral populations (*K*), we selected the *K* value presenting the highest rate of change in the log probability of data, following Evanno, Regnaut, and Goudet (2005). The congruence of the different runs for this *K* value was checked, and the run with the highest log probability was retained. For further analysis, samples whose estimated proportion of genome originating from one population (*q*, hereafter admixture coefficient) was below a 0.8 threshold were considered as resulting from admixture between populations. Individuals with a *q* value equal or superior to 0.8 for a given population were assigned to that population. We also used the TESS v2.3.1 software (Chen, Durand, Forbes, & François, 2007) to test whether results of this clustering algorithm converged with those of STRUCTURE.

For sorghum, whose genetic diversity showed a clear geographic pattern, further analyses were conducted on local varieties (160 samples), using those plants that could be assigned to a population (*q* ≥ 0.8). Isolation by distance among sites was tested with a Mantel test assessing the correlation between pairwise Nei's genetic distances (Nei, 1972) and log‐transformed geographic distances. This test was performed with 1,000 permutations, using the *mantel* function of the R package *ecodist* (Goslee & Urban, 2007). Then, a spatial principal component analysis (sPCA) was conducted to further detect and characterize the geographic patterns of diversity (Jombart, Devillard, Dufour, & Pontier, 2008). A discriminant analysis of principal components (DAPC) was also carried out to test whether genetic differences existed among the local varieties collected in the ten sites (Jombart, Devillard, & Balloux, 2010). Both analyses were performed using the *spca* and *dapc* functions implemented in the R package *adegenet* (Jombart, 2008). Analyses were conducted with R 3.2.3 (R Core Team 2015).

#### 2.5.3 Comparing sorghum genetic diversity in Mount Kenya and Africa

For sorghum, we conducted a joint analysis of our data set and a subset of 1,799 individuals from the collection of African landraces studied by Billot et al. (2013) with 41 SSRs. STRUCTURE was first run on the subset of 1,799 individuals from the African collection for *K* ranging from 1 to 20 populations. The resulting groups were then used as a priori to run DAPC, based on the 14 SSRs shared with the Mount Kenya data set. Group membership of Mount Kenya varieties was then predicted using the *predict.dapc* function.

## 3 Results

### 3.1 Varietal diversity patterns and diffusion pathways

A total of 20 pearl millet named varieties were inventoried across the ten sites, out of which 5 were exogenous varieties, 14 of local origin, and 1 of unknown origin. The main pattern arising from pairwise comparisons of variety‐based *F*_ST_ among sites is the clear differentiation of the varietal composition of site M1 (Table 1). Indeed, exogenous varieties (mainly *Kiraka* and *Ikira Sati*) were present in most sites and dominant in their portfolio (62% in average over sites, *SD* = 18%—Fig. S1), except in site M1. Furthermore, significant differences of composition among some pairs of sites reflected the uneven distribution of the main local variety, *Ciakaungi,* which was absent from the southernmost (M1) and northernmost sites (L4, M5, M6).

**Table 1:** Pairwise variety name‐based *F*_ST_ (Weir and Cockerham) values among the 10 sites where varieties were inventoried

Pearl millet	L2	L3	L4	M1	M2	M3	M4	M5	M6
L1	0.10	0.02	0.01	0.27**	−0.01	0.01	0.05	0.07	0.03
L2		0.07	0.15*	0.11*	0.04	0.11	0.02	0.04*	0.09
L3			0.01	0.15**	0.01	0.06	0.06	0.02	0.08*
L4				0.28*	0.05	0.12	0.13**	0.07	0.06
M1					0.18*	0.29***	0.18**	0.08*	0.26**
M2						−0.01	0.01	0.05	0.04
M3							0.04	0.13**	0.10**
M4								0.05	0.05
M5									0.08

Sorghum	L2	L3	L4	M1	M2	M3	M4	M5	M6
L1	0.05	0.02	0.01	0.10***	0.01	0.05**	0.09***	0.15***	0.02
L2		0.15**	0.13***	0.13***	0.10***	0.11***	0.11***	0.19***	0.12**
L3			−0.02	0.16***	0.01	0.08**	0.14***	0.19***	0.01
L4				0.16***	0.01	0.07*	0.14***	0.19***	−0.00
M1					0.08***	0.11***	0.10***	0.14***	0.15***
M2						0.05**	0.1***	0.14***	0.02
M3							0.01	0.03	0.03
M4								0.02	0.09
M5									0.13*

*p < .05; **p < .01; ***p < .001.

For sorghum, a total of 30 named varieties were inventoried, 10 of them were exogenous, mostly released by the formal improvement system (all were single‐season) and 18 were local (16 single‐season and 2 ratoon), the other 2 being of unknown origin. Differences of varietal composition were observed among sites for this species. Two major groups emerged from the pairwise comparisons of variety‐based *F*_ST_ among sites (Table 1). The first group was composed of sites M3, M4, M5, and the second one of sites L1, L3, L4, M2, and M6. In addition, site M1 differed significantly from all other sites, and it was also the case for site L2, except that it was not significantly different from site L1. Indeed, the local varieties were unevenly distributed among sites. Single‐season sorghum varieties were less abundant in sites M3, M4, and M5 (*M* = 61 %, *SD* = 5%), where ratoon varieties (mostly *Mugana*) were grown. In the other sites, farmers grew mostly single‐season varieties, but differences existed among sites, L1, L3, L4, and M2 showing similar patterns in cultivating *Mugeta* while farmers in site L2 grew mainly *Mucarama* and those of site M1 grew *Gatururu*. Furthermore, exogenous varieties (mainly *Kaguru*) were present in most sites, but their proportion was lower for sorghum than for pearl millet (44% in average, *SD* = 12%—Fig. S1).

The analysis of the sources of October 2006 seed lots showed that they were mainly self‐produced for both species, with averages of 56 and 54% for pearl millet and sorghum over the 10 sites (Table 2). Local markets were also important seed sources (pearl millet: 24%; sorghum: 22%), as well as external institutions such as government, NGOs, or church for sorghum (16%). Markets where farmers buy most of their seeds were located at less than 10 km from their home. Sites L1 and L2, M3 and M4 were linked to the same lowland market, but sites M3 and M4 were also linked to a highland market. Site L3 and L4 were linked to one common lowland market but also to separate ones. Sites M1, M2, M5, and M6 were linked to distinct markets, but farmers in the two latter sites, respectively, reported buying seeds in the Tigania and Tharaka areas.

**Table 2:** Seed lots sources

Sources	Pearl millet	Sorghum
2006 seed lots (%)		
Own seed	56 (16)	53 (14)
Market	25 (12)	22 (7)
Relative	8 (9)	5 (4)
Friend	5 (8)	4 (4)
Others	6 (7)	16 (10)
Initial seed lots (%)		
Relative	32 (26)	33 (16)
Market	33 (24)	21 (14)
Friend	3 (6)	2 (2)
Others	32 (20)	44 (12)

Mean percentage of October 2006 and initial seed lots obtained from the different sources over the ten sites. Standard deviation among sites indicated between parentheses.

Significant differences in the proportion of the different seed sources (self‐produced, obtained from relatives and friends vs. market and others) were observed for sorghum between exogenous and local varieties as local varieties were more self‐produced and obtained from relatives (Fisher's exact test: *p* < .05), but no such differences were observed for pearl millet (*p* = .143). For both species, seed sources did not differ significantly among sites (Fisher's exact test: *p* > .05).

The initial sources of seed lots were often forgotten by farmers. Otherwise, seeds had mainly been obtained initially from exogenous institutions, such as extension services, NGOs, church, or government administration (Table 2, pearl millet: 32%, sorghum: 44%), from relatives (pearl millet: 32%, sorghum: 33%), and from market (pearl millet: 33%, sorghum: 21%). For both species, local varieties were more frequently obtained from relatives and friends than other sources. For sorghum, seed sources did not significantly differ among sites, whereas for pearl millet market importance was significantly lower in sites L2 and L4 than in sites M4 and M6 (pairwise Fisher's exact tests: *p* < .05).

### 3.2 Organization of sorghum and pearl millet genetic diversity on Mount Kenya

For pearl millet, there were no significant differences among sites for gene diversity (mean of 0.66; range from 0.63 to 0.70) and for unbiased allelic richness (mean of 5.22; range from 5.0 to 5.7; Table 3). Furthermore, observed heterozygosity was high and also very homogeneous among sites (mean of 0.65; range from 0.59 to 0.68). Genetic differentiation among sites was very low, with a global fixation index (*F*_ST_) value of 0.007 (*p* < .001) and only one significant pairwise *F*_ST_ value at the 5% threshold (between sites L1 and L3, *F*_ST_ = 0.02; Table S4). Results of STRUCTURE and TESS clustering algorithms converged to show an absence of structure in pearl millet genetic diversity on Mount Kenya (Fig. S2).

**Table 3:** Parameters of genetic polymorphism for pearl millet and sorghum

Species	Site	*N*	*H*_nb_	*H*_o_	*A*_a_	*A*_s_
Pearl millet	L1	30	0.67	0.65	61	5.1
	L2	27	0.66	0.67	65	5.3
	L3	30	0.63	0.64	66	5.2
	L4	30	0.64	0.63	59	5.0
	M1	19	0.70	0.66	60	5.4
	M2	31	0.65	0.68	61	5.0
	M3	23	0.68	0.66	62	5.2
	M4	34	0.69	0.67	73	5.7
	M5	30	0.66	0.62	64	5.1
	M6	32	0.65	0.59	68	5.2
Sorghum	L1	27	0.40	0.03	49	3.0
	L2	26	0.40	0.00	48	3.0
	L3	36	0.35	0.00	37	2.2
	L4	26	0.33	0.00	41	2.6
	M1	29	0.44	0.01	46	2.9
	M2	27	0.44	0.01	55	3.4
	M3	50	0.53	0.02	63	3.7
	M4	34	0.53	0.02	64	3.9
	M5	38	0.51	0.02	64	3.9
	M6	28	0.48	0.01	57	3.5

*N*, number of samples; *H*_nb_, unbiased gene diversity; *H*_o_, observed heterozygosity; *A*_a_, total number of alleles observed over the 10 loci (pearl millet) and 16 loci (sorghum), and *A*_s_, unbiased allelic richness computed for 11 samples per site for pearl millet and 23 samples for sorghum.

For sorghum, gene diversity and allelic richness were lower than for pearl millet. The mean gene diversity was of 0.44 (range from 0.34 to 0.53), but there were no significant differences among sites (*p* > .05). The mean unbiased allelic richness was 3.21 (range from 2.2 to 3.9). It was significantly lower in lowland sites L3 and L4 as compared to other sites and significantly higher in sites M4 and M5 as compared to lowland sites (*p* < .05). Observed heterozygosity (*H*_o_) was very low, in contrast to pearl millet, and it did not vary among sites (mean of 0.01, range from 0 to 0.03).

**Figure 3:**
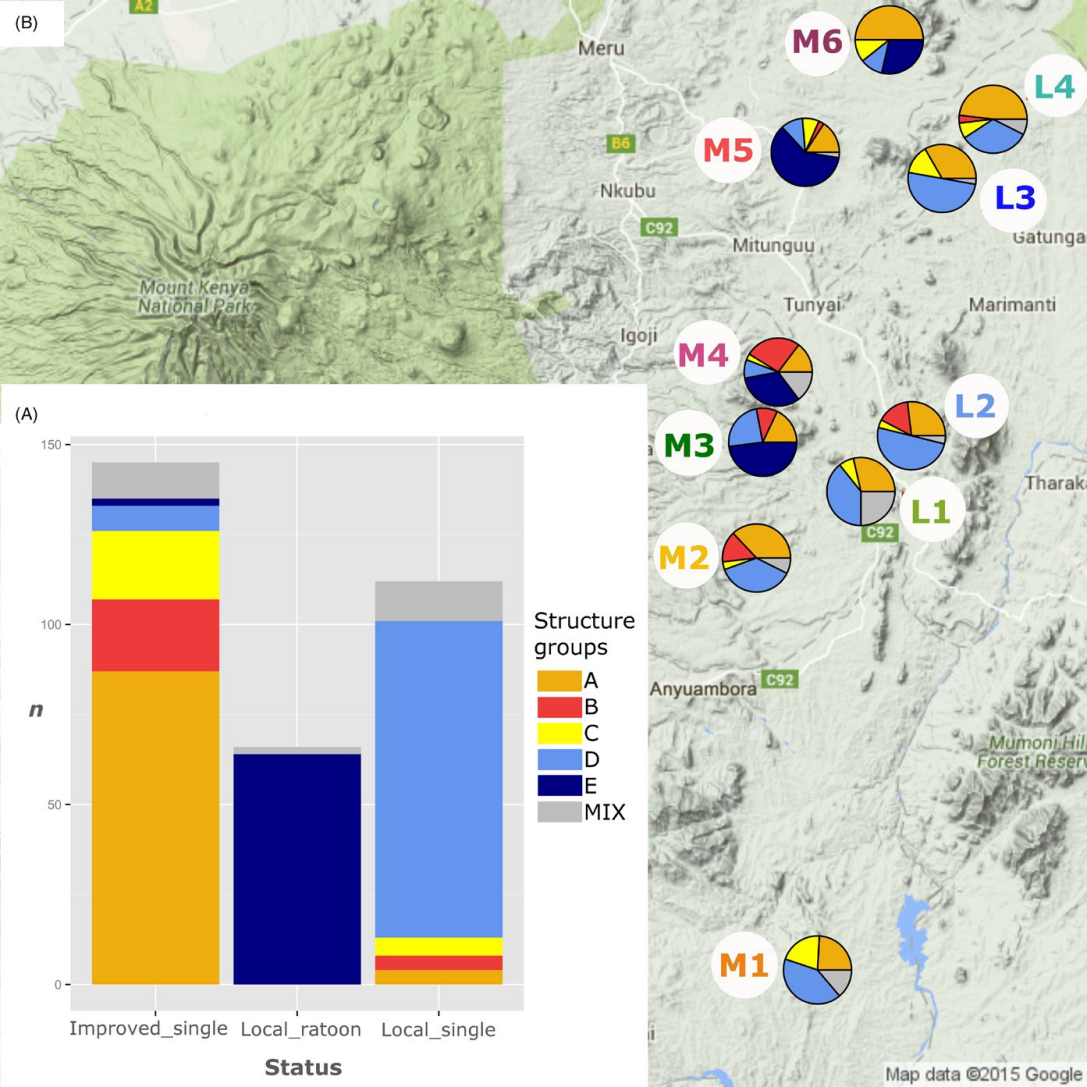
Structure and geographic patterns of sorghum genetic diversity. (a) Correspondence between the genetic groups inferred by STRUCTURE and varieties’ status (improvement and cycle). (b) Abundance of the five major genetic groups (STRUCTURE *K* = 5) in each site (*N* = 321 samples)

The global *F*_ST_ calculated for the ten sites was 0.10, indicating that genetic differentiation existed among sites for sorghum. Pairwise *F*_ST_ values varied significantly, ranging from 0 to 0.27 (Table 4). No significant genetic differentiation was detected among lowland populations, but these were significantly different from the midland populations of sites M3, M4, M5, and, to a lesser extent, from that of site M6. Among midland populations, the southernmost sites M1 and M2 were different from M3, M4, and M5, themselves being different from the northernmost site M6. A plausible population number according to Evanno's criteria was *K *=* *5, value for which STRUCTURE runs were highly congruent and the proportion of admixed individuals was low (6.8% of samples had a *q* admixture coefficient under 0.8). The groups inferred by STRUCTURE corresponded to those identified using TESS, and they matched with the information on the local or exogenous status of the samples, their growth cycle length, as well as with variety names. Three distinct genetic groups clustered exogenous varieties, corresponding, respectively, to *Kaguru* (A)*, Seredo,* and *Serena* (B)*,* and to varieties bearing various names (C). The two other groups clustered local varieties corresponding, respectively, to single‐season varieties (D), mainly *Mugeta* and *Mucarama*, and to ratoon varieties (E), mainly *Mugana (*Fig. 3a).

**Table 4:** Pairwise allele‐based *F*_ST_ (Weir and Cockerham) values among the sorghum populations collected in the 10 sites

	Lowlands			Midlands					
	L2	L3	L4	M1	M2	M3	M4	M5	M6
Lowlands									
L1	0.00	0.00	0.01	0.03	0.00	0.13**	0.10**	0.22**	0.08
L2		0.01	0.05	0.03	0.00	0.12**	0.09**	0.22**	0.11**
L3			0.03	0.04	0.02	0.18**	0.15**	0.27**	0.12**
L4				0.05	0.02	0.18**	0.15**	0.26**	0.05
Midlands									
M1					0.02	0.13**	0.09**	0.20**	0.07
M2						0.11**	0.08*	0.19**	0.06
M3							0.01	0.04	0.09**
M4								0.05	0.07*
M5									0.11*

*p < .05; **p < .01.

The relative abundance of the different genetic groups inferred by STRUCTURE for sorghum varied strongly among sites (Fig. 3b). Exogenous gene pools (clusters A, B, C) were especially less abundant in midland sites M3 and M5 than in the others. The genetic group corresponding to ratoon landraces (cluster E) was abundant in sites M3–M6 while absent in the lowlands and in the southernmost midland sites M1 and M2. Conversely, the local single‐season genetic group (D) was abundant in lowland sites L1–L4 and in midland sites M1 and M2. This geographic distribution of genetic clusters is congruent with the *F*_ST_ differentiation matrix.

**Figure 4:**
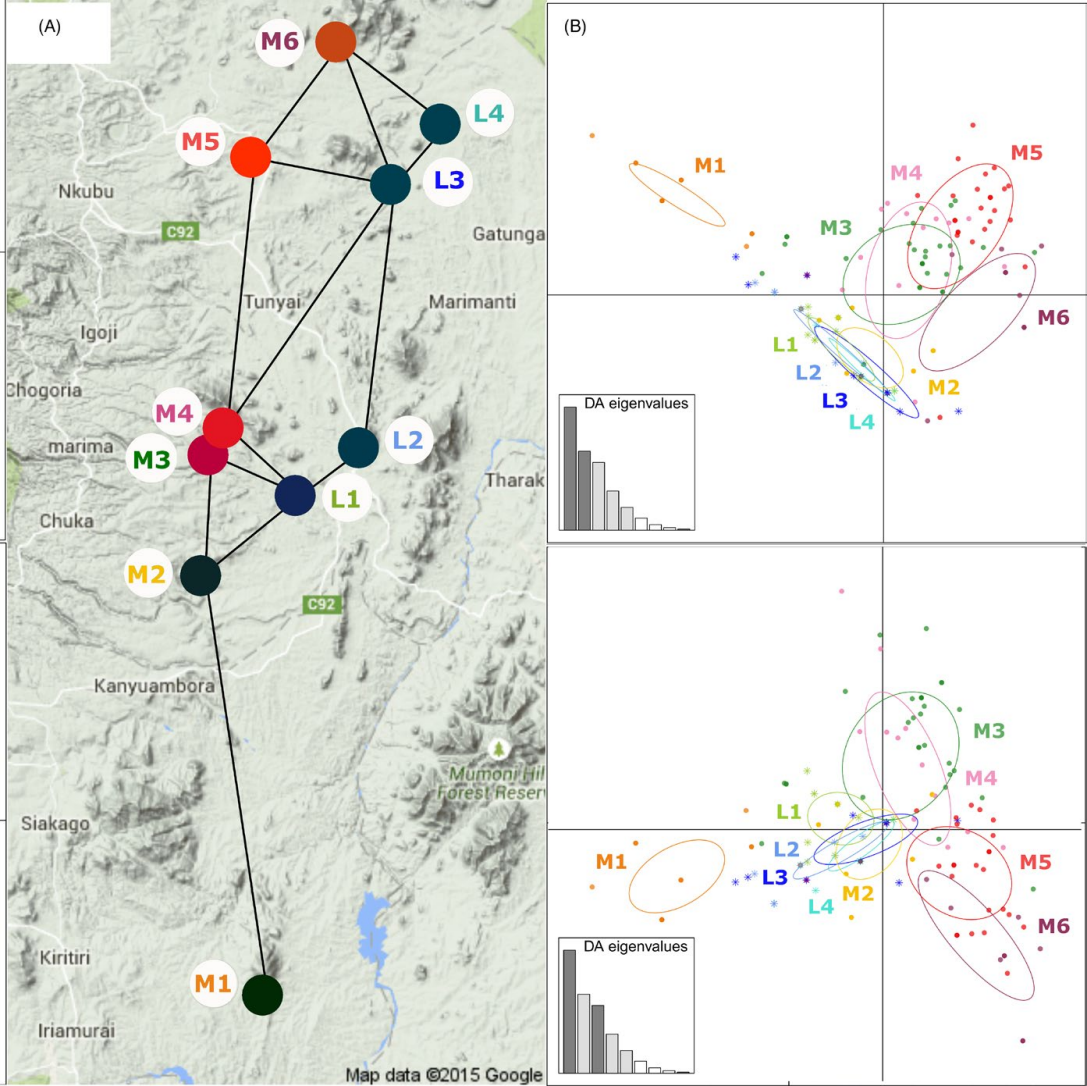
Geographic patterns of sorghum local genetic diversity in the Mount Kenya region. (a) Projection of the color plot synthesizing the sorghum populations (sites) coordinates on the first three sPCA components (local genepool as assigned by STRUCTURE,* N* = 160 samples). Each principal component is represented as gradient of a given color channel; the first PC is shown in red, the second PC in green, and the third PC in blue. (b) Plot of samples’ scores on DAPC first discriminant axis (top: DA 1 and 2; bottom: DA 1 and 3). Lowland sites are symbolized by stars and midland sites by dots

Further analysis within the local pool (STRUCTURE groups D and E) enabled us to discern geographic patterns in the distribution of indigenous sorghum genetic diversity as shown by the significant Monte Carlo test for global structure (*p* = .03) in the sPCA. These patterns were not determined by mechanisms of isolation by distance, as the Mantel test showed no relationship between pairwise log‐transformed geographic distances and genetic distances among the 10 sites (Mantel's *r* = .048, *p* = .33). Instead, the geographic patterns displayed by mapping the three first scores of the sPCA were congruent with DAPC scatter plots (axis 1–3). Both analyses revealed the genetic differentiation of midland sites M3, M4, M5, and M6, where ratoon varieties were grown, from the other sites (Fig. 4). Furthermore, the third sPCA principal component and the first DAPC component highlighted the genetic differentiation of the southernmost site M1 from all other sites. DAPC scatter plots further suggested that a gradual genetic differentiation of the local genetic pool exists between the south and the north of the midland transect.

### 3.3 Comparing sorghum genetic diversity in Mount Kenya and Africa

**Figure 5:**
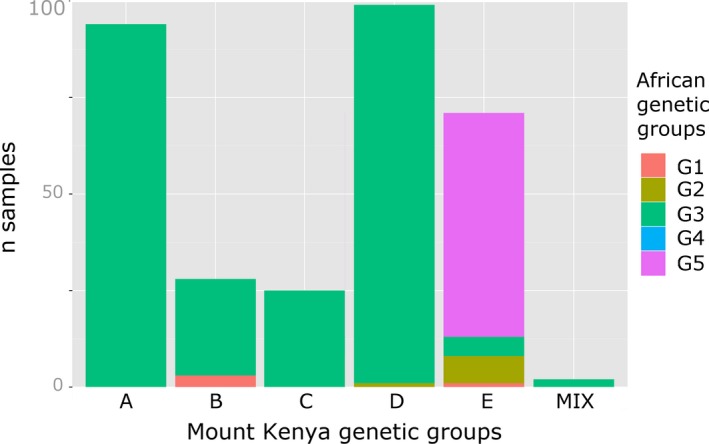
DAPC genetic assignment of Mount Kenya sorghum samples (*N* = 321) in the five African genetic groups inferred using STRUCTURE (*N* = 1,799). G1: durra, bicolor, and intermediates from northeastern Africa; G2: kafir and intermediates from southern Africa; G3: caudatum and intermediates from Central and East Africa; G4: guinea and intermediates from West Africa; G5: caudatum, guinea, and intermediates from southeastern and Central Africa

According to STRUCTURE outputs, *K *=* *5 was found to be an accurate number of clusters to describe the organization of African landrace genetic diversity. A clear decrease in log‐likelihood was observed for this number of populations and it provided a more precise description of diversity than the *K *=* *2 solution identified with the Evanno criterion. The five clusters inferred at the continental scale corresponded to distinct cultivated sorghum races and geographic origins (Fig. S3). Thus, genetic cluster 1 corresponded to durra, bicolor, and intermediates from northeastern Africa, group 2 to kafir and intermediates (mainly kafir–caudatum and guinea–caudatum) from southern Africa, group 3 to caudatum and intermediates (mainly guinea–caudatum) from Central and East Africa, group 4 to guinea and intermediates (durra–caudatum and guinea–caudatum) from West Africa, and group 5 to caudatum, guinea, and intermediates between them from southeastern and Central Africa (extended Great Lakes region). Ratoon and single‐season varieties from Mount Kenya were assigned to two different groups according to DAPC outputs. The ratoon landraces were assigned to the group 5 while both local and exogenous single‐season varieties were assigned to the group 3 (Fig. 5). These results thus indicated that ratoon and single‐season sorghum varieties were introduced in Mount Kenya region from different regions.

## 4 Discussion

In this study, we analyzed the dynamics of sorghum and pearl millet varietal and genetic diversity on Mount Kenya. Differences of diversity patterns were observed between these two species at the variety level, based on vernacular names, and at the genetic level. Pearl millet varietal composition was fairly uniform among sites, except for site M1. Indeed, two exogenous varieties were abundant and presented a wide geographic distribution, which partially blurred the distribution patterns of local varieties. At the genetic level, no differentiation was detected among the pearl millet populations grown in the different sites, which suggests a very high level of co‐ancestry and/or genetic connectivity among them. Furthermore, the absence of genetic differences between local and exogenous varieties for this species illustrates the effect of strong pollen‐mediated gene flow, which contributes to the genetic homogenization at the region level.

For sorghum, geographic patterns of varietal and allelic compositions were strongly related. Even though exogenous varieties were also widespread in the region for this species, they were less abundant than for pearl millet and little admixture was observed with local gene pools. Patterns resulting from the geographic distribution of local varieties thus clearly arose. Ratoon varieties, genetically distinct from single‐season ones, were grown exclusively in part of the sites (M3–M6). Among the sites growing only single‐season local varieties, site M1 presented significantly different varietal and genetic compositions. These patterns seem to result from the combination of processes at different spatiotemporal scales. First, the coexistence of distinct genetic groups in the area suggests the contribution of gene pools from different origins, which was confirmed by comparing Mount Kenya genetic diversity with that of the African continent. Second, our results show that barriers to gene flow exist among sites (restricted seed‐mediated gene flow) and within sites (restricted pollen‐mediated gene flow), which maintain the diversity structure and geographic patterns observed.

To improve our understanding of the diversity patterns observed, we investigated the seed‐mediated gene flows through the survey of local seed systems. Seed sources were found to be similar for sorghum and pearl millet, with a major importance of local sources as seeds were mostly self‐produced and bought on local markets, usually located at less than 10 km. Interestingly, the geographic structure of seed systems did not limit pearl millet genetic homogenization on Mount Kenya, while it limited that of sorghum. This situation shows that the combination of wide diffusion of planting material and intense pollen‐mediated gene flow plays a major role in blurring genetic differences among cultivated populations at a regional scale. Our results thus illustrate the complex interplay between crop breeding systems, which determine pollen‐mediated gene flow, and the multiple processes involved in shaping seed‐mediated gene flow on different timescales, which are further discussed hereafter.

### 4.1 Large diffusion of exogenous varieties and intense gene flow homogenized pearl diversity

At the Mount Kenya scale, patterns of pearl millet varietal and genetic diversity suggest that the combination of both high pollen‐mediated gene flow among varieties and seed‐mediated gene flow by the mean of a large diffusion and adoption of exogenous varieties drove genetic homogenization. Indeed, pearl millet genetic diversity showed no structure, neither at the region nor at the site scales, and no genetic differences were detected between indigenous and exogenous or between different named varieties grown at a same site.

Despite the limited geographic extent of local seed systems, the exogenous pearl millet varieties *Kiraka* and *Ikira Sati* were largely grown in most sites. *Kiraka* was introduced in the area in the 1930s from West Africa by a missionary according to elder farmers (BR personal observation), while *Ikira Sati* was released under the name ICMV 221 by ICRISAT India and was bred from West African material (Witcombe, Rao, Raj, & Hash, 1997). Because of their large diffusion over time across the eastern slope of Mount Kenya and because of their crossing with the local preexisting varieties, these exogenous varieties may thus have acted as a “genetic bridge” and contributed to homogenize the local pearl millet varieties across sites. As pearl millet is strongly outcrossing, pollen‐mediated gene flow within and among neighboring fields should occur, and crosses among varieties are expected to be frequent because they are usually mixed in the same field (personal observation). Intense pollen‐mediated gene flow thus likely contributes to the absence of genetic structure in the Mount Kenya region.

Our results coincide with other studies conducted on pearl millet in different areas. Such an absence of genetic structure was observed at the village scale by Busso et al. (2000) in northeastern Nigeria, showing that pearl millet varieties bearing different names could not be distinguished with neutral markers. On a region scale, the study of vom Brocke et al. (2003) in western Rajasthan (India) showed that intense seed exchanges homogenized pearl millet populations. Studies in West Africa, however, reported genetic patterns resulting from more constrained seed diffusion of local varieties, which somehow differ from our case study. For instance, genetic differences among the pearl millet populations of the same variety grown in different villages were observed, seed exchanges being rare among farmers (Busso et al., 2000). The effect of seed diffusion pathways on pearl millet diversity was also observed by Allinne et al. (2008) in the Zarma region of Niger. They suggested that asymmetrical seed flows from southern regions with higher rainfall to northern ones induced a significant albeit weak genetic differentiation between these two areas.

A number of studies on other open‐pollinated crop species, especially on maize, reported the homogenizing action of pollen‐mediated gene flow on populations. Based on simulations conducted with a crop metapopulation model on this species, van Heerwaarden, van Eeuwijk, and Ross‐Ibarra (2010) showed that “high levels of pollen migration may mask the effects of seed management on structure.” Furthermore, empirical studies largely described the process of creolization (exogenous improved varieties becoming genetically similar to local ones due to selection of seeds for replanting in new local conditions and their hybridization with local landraces) for maize, in Mexico (Bellon, Adato, Becerril, & Mindek, 2006) and Tanzania (Westengen, Ring, Berg, & Brysting, 2014). However, restricted seed‐mediated gene flow may also conduct to genetic differentiation of populations, as was observed on maize among adjacent ethnolinguistic groups in Mexico (Orozco‐Ramírez et al., 2016).

### 4.2 Multiple diffusions and restricted gene flow produce regional patterns of sorghum diversity

For sorghum, our results suggest that different gene pools conveyed to the Mount Kenya region through diverse past human migrations on the one hand, and through more recent introductions by the formal improvement and diffusion system on the other hand. Then, the combination of limited pollen‐mediated gene flow on a local scale and restricted diffusion of local varieties on a regional scale contributed to maintain the genetic structure and geographic patterns observed.

#### 4.2.1 Multiple diffusion origins

The five different genepools for sorghum coexisting on Mount Kenya reflect their different ancestry. Three of them were released by the formal varietal improvement system, information available for part of them showed that they were bred from East African material (Doggett, 1988), and comparison with the African continent diversity confirmed that they mostly relate to the caudatum and intermediates group from Central and East Africa. The two local genepools (ratoon and single‐season), however, relate to different groups as single‐season varieties relate to the caudatum and intermediates pool of Central and East Africa while ratoon varieties rather relate to the caudatum, guinea, and intermediates cultivated in the Great Lakes area and southeastern Africa. These results coincide with those of previous studies, showing that Mount Kenya ratoon varieties (*Mugana*) were genetically distinct from sorghum grown in the rest of Kenya, while local single‐season varieties (*Mugeta* and *Mucarama*) clustered with accessions collected in different other regions of Kenya (Mutegi et al., 2011). Results of our study and of previous ones thus converge to suggest that ratoon and single‐season varieties have different geographic origins and followed different diffusion pathways.

Such genetic proximity between Mount Kenya ratoon varieties and accessions collected in the Great Lakes region coincides with collection reports showing the importance of ratoon varieties in Uganda, especially in western Uganda where they are the only sorghum type cultivated (Esele & Maxted, 1985). This genetic proximity suggests past contacts among human populations of the Great Lakes and those of Mount Kenya and raises questions concerning processes involved. Complex migratory and commercial patterns in these areas, and recent replacement of local varieties by exogenous ones, make it difficult to reconstruct the history of ratoon sorghum on Mount Kenya. The coincidence between their distribution area and that of Meru ethnolinguistic unit pleads for an introduction following the migration of this human group. One hypothesis could be that these ratoon varieties were brought from the Great Lakes area to the Kenyan coast through Swahili trading activities, and later brought to Mount Kenya by the Meru people while fleeing Arabs invasions around the 18th century (Middleton & Kershaw, 1965), but this deserves further investigations.

#### 4.2.2 Restricted seed‐mediated and pollen‐mediated gene flow

The combination of limited pollen‐mediated gene flow on a local scale and restricted seed‐mediated gene flow among geographic areas is also involved in the diversity patterns observed for sorghum. At a local scale, limited pollen‐mediated gene flow impedes the genetic homogenization among varieties. Indeed, sorghum is mainly selfing even though outcrossing rates were found to vary between 5 and 40 % (Barnaud, Trigueros, McKey, & Joly, 2008; Chantereau & Kondombo, 1991; Ollitrault, Noyer, Chantereau, & Glaszmann, 1997). Limited pollen‐mediated gene flow is reflected by the genetic differences among the five pools although they were frequently found in the same sites and farms. Outcrossing rate of the sorghum populations studied was not measured, and may vary among the different genetic groups. Contrarily to pearl millet, genetic differences were detected between local and exogenous varieties. Differences also exist among exogenous varieties, as well as between local single‐season and ratoon varieties for which asynchrony of flowering should contribute to limit crosses. Similar genetic differences among sorghum varieties on a local scale were described in many studies, resulting from limited pollen‐mediated gene flow combined to farmers’ conservative selection practices. For instance, genetic differences were found in northern Cameroon between varieties grown on a village scale (Barnaud et al., 2007), and limited creolization was observed by Rabbi et al. (2010) in western Kenya and eastern Sudan, and by Deu et al. (2014) in Mali.

At the Mount Kenya scale, patterns of sorghum varietal and genetic diversity indicate that seed diffusion of local varieties is constrained among geographic areas. Precisely, the diffusion of local ratoon varieties is limited between the northern midland sites (M3–M6) and the rest of the region, while the reverse is observed for the diffusion of local single‐season varieties. In addition, site M1 also appeared isolated from the rest of the sites as it presented different varietal and genetic compositions. Such connectivity patterns can result from the combination of multiple factors, which are discussed hereafter. On a region or country scales, most studies on sorghum genetic diversity also reported the existence of a strong geographic structure, for instance in Niger (Deu et al., 2008), Zambia (Ng'uni, Geleta, & Bryngelsson, 2011), and Kenya (Mutegi et al., 2011). Such patterns resulted from limited seed‐mediated gene flow among areas, and multiple drivers of social, economic, or ecological origins were evoked in these studies to explain such restrictions of seed diffusion.

### 4.3 Factors shaping seed diffusion on Mount Kenya

In the Mount Kenya region, the contribution of social, economic, and ecological factors is difficult to disentangle as the observed picture can result from multiple interactions (Sagnard et al., 2008). We review here the mechanisms potentially involved in determining farmers’ seed choices, and we distinguish processes related to the availability and accessibility of planting material for farmers on the one hand, and those related to its adaptation to their needs on the other hand (Sperling, Cooper, & Remington, 2008).

#### 4.3.1 Availability and accessibility

Variety availability and accessibility to farmers is largely determined by the geographic distance to the various seed sources and by their social networks. On Mount Kenya, local seed systems were found to be of limited geographic extent, except for exogenous varieties, which were widely distributed by the extension services and NGOs. Our survey showed that local sorghum and pearl millet varieties were produced, diffused, and cultivated within a limited geographic area. Indeed, seeds of local varieties were self‐produced, obtained from relatives and friends, or purchased at the local market. This means that the seed lots of local varieties sown by farmers were mostly produced locally (within ca. 10 km). Such a limited extent of local seed systems was frequently observed (Bellon et al., 2011; Hodgkin et al., 2007). Local markets are an important seed source for small farmers, as was reported in an extensive multicrop study in Central and East Africa (McGuire & Sperling, 2016), so they can affect the organization of crop genetic diversity. For instance, studying barley in Ethiopia, Samberg et al. (2013) concluded that marketed varieties present a weaker geographic structure as compared to nonmarketed ones.

Access to the different varieties is also influenced by social organization. In the Mount Kenya region, recent studies showed that social relationships were involved in seed diffusion as exchanges occurred mainly among relatives, being thus limited among ethnolinguistic units (Labeyrie et al., 2014, 2016). Indeed, in the present study too, we observed a fairly good match between the geographic patterns of sorghum local genetic and ethnolinguistic diversity. Farmers located in the Chuka–Tharaka ethnolinguistic unit (M2 and L1–L4) grow mainly local single‐season varieties, while farmers located in the Meru unit (sites M5 and M6) grow mainly ratoon varieties. Sites located in the transitional area between these two ethnolinguistic units (M3 and M4) grow both ratoon and single‐season varieties. Lastly, the single‐season varieties in the Mbeere ethnolinguistic unit (site M1) are genetically different from those grown in the rest of the area. Such a dependence of seed diffusion networks on farmers’ social organization was described for various crop species in different societies (Coomes et al., 2015).

The distance between farms and markets also contributes to determine the planting material farmers can access. Interestingly, the location of markets where farmers from different study sites purchase their seeds appeared to be related to the proportion of ratoon and single‐season sorghum varieties in their portfolio. First, ratoon varieties were absent in the sites that depend exclusively on lowland markets (L1–L4), where they were not sold (personal observation). Secondly, farmers in the southern midlands (M1, M2), growing only single‐season varieties, and those in the northern midlands (M5, M6), growing mainly ratoon local varieties, purchase seeds in markets located in their respective areas. Last, farmers in central midlands (M3 and M4), who grow both local single‐season and ratoon varieties, purchase seeds in both lowlands (where single‐season varieties are sold) and highland markets (where ratoon varieties are sold). However, this relationship between the varieties grown by farmers in the different sites and those sold on the markets is bidirectional: Farmers buy seeds that are available and accessible in the closer markets, but conversely markets have to respond to farmers needs in terms of use and adaptation.

#### 4.3.2 Adaptation

Varieties obtained by farmers either directly from other farmers or on the markets are expected to be adapted to their needs in terms of agronomic, organoleptic, and other characteristics (Bellon, 1996). Criteria of seed choice can be sociocultural, economic, and agroecological (Bellon et al., 2011; Stromberg, Pascual, & Bellon, 2010). In the present case, potential differences of culinary uses of the ratoon and single‐season varieties among ethnolinguistic units may be involved (Linsig, 2009), but these aspects remain poorly documented. Specific uses or cultural value associated with the ratoon by the Meru farmers could thus contribute to explain their common geographic distribution.

Furthermore, adaptation of varieties to different agroecosystems is also potentially a major factor influencing seed diffusion networks, as discussed by Bellon et al. (2011) for maize in Mexico and Samberg et al. (2013) for barley in Ethiopia. This does not seem to be the case for pearl millet on Mount Kenya, as the distribution of varieties and genetic diversity showed no relationship with the climatic gradient along its slopes. In contrast, the distribution of sorghum ratoon and single‐season varieties matched partially the agroecological zones defined by Jaetzold et al. (2007). These different varieties presented different agronomic characteristics, ratoon varieties requiring a longer time to complete their growth cycle and being grown during two cropping seasons instead of one for the single‐season varieties. Such a longer flowering time could result from adaptation to higher elevations where growing seasons are longer. Indeed, ratoon varieties were dominant in the northern midlands area, presenting a cooler and more humid climate while single‐season varieties were dominant in the semiarid lowlands and southern midlands. Little information concerning the climatic adaptation of ratoon sorghum is available, but the lax panicle which is one characteristic of Mount Kenya ratoons could be an adaptation to humid climate (De Wet, 1978; Mekbib, 2008). Last, the geographic distribution of ratoon sorghum also corresponded to differences in cropping systems. They were the major sorghum varieties grown in the northern midlands, where cropping systems are more diversified and include a larger share of cash crops, while they were not grown in the lowlands and southern midlands where agroecosystems are dominated by sorghum and pearl millet, grown mainly for self‐consumption within large fields. Differences of adaptation, uses, and role of the ratoon and single‐season varieties in the different agroecosystems could thus contribute to explain their restricted geographic distribution.

#### 4.3.3 Implications for genetic resources conservation

Our study enabled to highlight clear geographic patterns of genetic diversity for sorghum. Ratoon varieties are of special interest for conservation, as they are genetically different from the rest of the Kenyan populations, being related to Great Lakes populations from which they evolved separately. Their uneven distribution suggests that they present particular characteristics, either related to agronomic and climatic adaptation to high‐altitude agroecosystems, or related to cultural preferences of the ethnolinguistic groups that grow them. These varieties are potentially at risk of extinction because of their agronomic and processing properties (longer growth cycle, time‐consuming dehulling), and the overall preferences of consumers for maize (Leclerc, Mwongera, Camberlin, & Moron, 2014). Based on the results of our study, targeted incentives and programs for *in situ* conservation of ratoon varieties should thus be designed.

## 5 Conclusion and Perspectives

This study depicted the complexity of the processes involved in sorghum and pearl millet diversity dynamics on the eastern side of Mount Kenya. Using landscape approaches, we showed that pollen‐mediated and seed‐mediated gene flow had a major impact on diversity patterns. It further showed that seed‐mediated gene flow on Mount Kenya is of limited extent and present geographic patterns, potentially influenced by distance between seed sources and users, by ethnolinguistic organization, and by agroecological constraints. Furthermore, this study points out that historic dynamics, associated with past human migrations, left their imprints in current crop diversity and should thus be investigated, even to apprehend local‐scale patterns.

Metapopulation models developed for crops may help to further investigate the determinants of sorghum and pearl millet genetic diversity patterns on Mount Kenya (van Heerwaarden et al., 2010). They would especially isolate the effects of pollen‐ and seed‐mediated gene flow, and assess demographic effects due to seed management and cropping system characteristics. Applying such models would require additional data collection, particularly concerning seed management practices such as the quantity and frequency of seed migration. Furthermore, defining values for some model parameters would raise methodological questions. For instance, estimating the pollen migration parameter for sorghum would be challenging as it was reported to vary strongly among varieties, regions, and years (Barnaud et al., 2008; Ellstrand, 1992).

Several authors have called for the development of landscape approaches in the framework of on‐farm conservation (Bellon, Gotor, & Caracciolo, 2015; Manel et al., 2003; Samberg et al., 2013). Indeed, these approaches help identifying factors that drive crop diversity evolution in smallholder farming systems. Our results support this idea and suggest that landscape genetics studies could gain from a comparative approach on several crop species. Such a comparison especially contributes in elucidating the relative contributions of social and biological factors in crop diversity patterns.

By showing the multiplicity of processes involved at different timescales in crop diversity dynamics, this study has important implications for the conservation of crop genetic resources. It showed that crop diversity dynamics are not exclusively driven by species’ biology and ecological processes. Farmer practices, notably concerning seed diffusion, have a major impact on these dynamics, but their determinants remain largely unknown. Our results call for a better understanding of factors shaping local seed systems, especially concerning the role of local markets on seed diffusion and thus on crop diversity patterns.

## Data Archiving Statement

Data available from the Dryad Digital Repository: http://dx.doi.org/10.5061/dryad.5g129.

## Supporting information


**Table S1**. Genetic sampling summary.
**Table S2**. Number of samples collected for each sorghum and pearl millet named variety and number of farms where they were collected (between brackets) for each site.
**Table S3**. Summary of information and genetic diversity estimates per locus.
**Table S4**. Pairwise *FST* values between sites for pearl millet
**Figure S1**. Distribution of the major pearl millet (N = 238 variety‐farm) and sorghum (N = 367 variety‐farm) named varieties among sites.
**Figure S2**. Results of STRUCTURE assignment for pearl millet (A., *N* = 286, *K* = 2), and sorghum (B., *N* = 321, *K* =5).
**Figure S3**. DAPC scatterplot (DA 1 and 2) of the five African genetic groups inferred using STRUCTURE (*N* = 1799).
