# Supplementary material for: Past and present dynamics of sorghum and pearl millet diversity in Mount Kenya region
Source: Evol Appl. 2016 Sep 23;9(10):1241–57. doi: 10.1111/eva.12405 (PMC5108216; doi:10.1111/eva.12405)

Supplementary materials

Table S1. Genetic sampling summary. Number of farms where the samples were collected (*N farms)*, number of named varieties collected (*N varieties)*, number of individuals of each variety collected per farm (*N samples/ farm/variety)*, total number of samples collected in each site (*N samples)***.**

	**Lowlands**				**Midlands**					
	**L1**	**L2**	**L3**	**L4**	**M1**	**M2**	**M3**	**M4**	**M5**	**M6**
**Pearl Millet**										
N farms	13	13	13	14	5	9	12	14	12	14
N varieties (total)	4	6	4	3	4	4	4	5	7	3
N samples/farm/variety	2	2	2	2	3	3	2	2	2	2
N samples (total)	30	27	30	30	19	31	23	34	30	32
**Sorghum**										
N farms	15	15	12	13	11	13	13	16	13	9
N varieties (total)	8	7	5	4	8	7	7	7	4	3
N samples/farm/variety	1	1	2	1	2	1	2	1	2	2
N samples (total)	27	26	36	26	29	27	50	34	38	28

Table S2. Number of samples collected for each sorghum and pearl millet named variety and number of farms where they were collected (between brackets) **for each site.**

	Lowlands				Midlands						
**Species**	**L1**	**L2**	**L3**	**L4**	**M1**	**M2**	**M3**	**M4**	**M5**	**M6**	**Total**
**Pearl millet**											
**Local**											
Ciakaungi	4 (2)	4 (2)	4 (2)	-	-	9 (3)	8 (4)	8 (4)	-	-	37 (17)
Mututwa	-	12 (6)	2 (1)	-	-	3 (1)	-	8 (4)	2 (1)	-	27 (13)
Kimeru	-	-	-	-	-	-	-	8 (4)	1 (1)	6 (3)	15 (8)
Mugombe	-	-	-	-	6 (2)	-	1 (1)	-	-	-	7 (3)
Gathingitu	-	-	-	-	-	3 (1)	-	-	-	-	3 (1)
Kimbeere	-	-	-	-	3 (1)	-	-	-	-	-	3 (1)
Mucena.	-	-	-	-	3 (1)	-	-	-	-	-	3 (1)
Gatiga g.	-	-	-	2 (1)	-	-	-	-	-	-	2 (1)
Mutiro Ig.	-	-	-	-	-	-	-	-	2 (1)	-	2 (1)
Mugimbi	-	-	-	-	-	-	-	-	1 (1)	-	1 (1)
Mwere	-	1 (1)	-	-	-	-	-	-	-	-	1 (1)
**Improved**											
Kiraka	20 (10)	4 (2)	12 (6)	20 (11)	-	13 (5)	12 (7)	8 (4)	8 (4)	22 (12)	122 (61)
Ikira Sati	4 (2)	4 (2)	12 (6)	8 (4)	7 (3)	3 (1)	2 (1)	2 (1)	14 (7)	4 (2)	57 (28)
Katumani	-	2 (1)	-	-	-	-	-	-	2 (1)	-	4 (2)
Ngirig.	2 (1)	-	-	-	-	-	-	-	-	-	2 (1)
**Sorghum**											
**Local single-season**											
Mugeta	7 (7)	1 (1)	18 (9)	9 (9)	2 (1)	9 (9)	10 (5)	5 (5)	4 (2)	6 (3)	71 (51)
Mucarama	5 (5)	11 (10)	-	-	-	-	2 (1)	3 (3)	-	-	21 (19)
Gatururu	-	-	-	-	9 (5)	-	-	-	-	-	9 (5)
Muruge	-	1 (1)	-	-	6 (3)	1 (1)	-	-	-	-	8 (5)
Mujeru	1 (1)	-	-	-	2 (1)	1 (1)	-	-	-	-	4 (3)
Karigu	-	-	2 (1)	-	-	-	-	-	-	-	2 (1)
Keritu	-	-	-	-	2 (1)	-	-	-	-	-	2 (1)
Kimbeere	-	-	-	-	2 (1)	-	-	-	-	-	2 (1)
Kitharaka	-	-	2 (1)	-	-	-	-	-	-	-	2 (1)
W.Mwere	-	-	-	-	-	-	-	2 (2)	-	-	2 (2)
Ciagak.	-	1 (1)	-	-	-	-	-	-	-	-	1 (1)
Mucuri	1 (1)	-	-	-	-	-	-	-	-	-	1 (1)
**Local ratoon**											
Mugana	-	-	-	-	-	-	18 (9)	12 (12)	24 (12)	6 (3)	60 (36)
Kathirigua	-	-	-	-	-	-	6 (3)	-	-	-	6 (3)

Improved

Kaguru	10 (10)	7 (7)	12 (6)	13 (13)	4 (2)	10 (10)	8 (4)	6 (6)	8 (4)	16 (8)	94 (70)
Seredo	-	4 (4)	2 (1)	2 (2)	-	2 (2)	2 (1)	1 (1)	-	-	13 (11)
Serena	-	1 (1)	-	-	-	-	4 (2)	5 (5)	2 (1)	-	12 (9)
Bunge	-	-	-	-	-	3 (3)	-	-	-	-	3 (3)
Mtama1	1 (1)	-	-	2 (2)	-	-	-	-	-	-	3 (3)
Ngirig.	1 (1)	-	-	-	2 (1)	-	-	-	-	-	3 (2)
Mukumbu	1 (1)	-	-	-	-	-	-	-	-	-	1 (1)
Nkuki Im.	-	-	-	-	-	1 (1)	-	-	-	-	1 (1)

Table S3. Summary of information and genetic diversity estimates per locus.
A^a: number of alleles and H_{nb}^{a}: Nei's unbiased gene diversity in this study
A^b: number of alleles and H_{nb}^{b}: Nei's unbiased gene diversity in the GCP collection of 1799 African sorghum landrace accessions

Species	SSR	[1]Ch / [2]LG	Repeat motif	Size range (bp)[a]	A^a	H_{nb}^a	Size range (bp)[b]	A^b	H_{nb}^b
**Pearl millet**									
	psmp2201	2	GT	328-363	5	0.58			
	psmp2202	5	GT	133-161	4	0.54			
	psmp2206	2	GT	195-205	6	0.73			
	psmp2249	NA	GT	118-151	7	0.65			
	psmp2247	NA	TG	193-205	7	0.41			
	psmp2085	4	AC	160-170	6	0.52			
	psmp2208	5	GT	208-336	20	0.69			
	psmp2203	7	GT	333-359	17	0.87			
	psmp2089	2	AC	102-138	17	0.84			
	psmp2266	7	GA	165-201	13	0.84			
**Sorghum**									
	sb4-72	6	AG	183-199	6	0.51	175-211	18	0.73
	sb5-206	9	AC/AG	106-152	16	0.79	100-164	32	0.95
	sb6-84	2	AG	183-207	9	0.55	171-235	30	0.83
	sbAGB02	7	AG	96-154	7	0.33	92-176	39	0.74
	Xcup02	9	GCA	192-204	4	0.64	186-207	8	0.61
	Xcup53	1	TTTA	186-195	3	0.49	182-202	9	0.42
	Xcup61	3	GAG	198-201	2	0.48	189-201	4	0.51
	Xtxp10	9	CT	131-151	7	0.8	133-155	12	0.75
	Xtxp40	7	GGA	126-138	3	0.02	108-144	12	0.24
	Xtxp114	3	AGG	208-214	3	0.35	196-223	10	0.52
	Xtxp136	5	GCA	240-243	2	0.5	240-246	3	0.38
	Xtxp145	6	AG	212-232	5	0.22	204-262	27	0.89
	Xtxp278	7	TTG	243-252	3	0.03	240-294	14	0.24
	Xtxp289	9	CCT/ AGG	286-333	16	0.78	NA	NA	NA
	Xtxp295	7	TC	161-185	10	0.76	NA	NA	NA
	gpsb123	8	AC/GA	288-298	6	0.74	284-304	11	0.75

[1] Ch: chromosomes numbered as per Kim et al. (2005) - Kim, J.S., P.E. Klein, R.R. Klein, H.J. Price, J.E. Mullet, and D.M. Stelly 2005. Chromosome identification and nomenclature of *Sorghum bicolor*. Genetics **169**:1169–1173.

[2] LG: linkage group on pearl millet genetic map

Table S4. Pairwise F_{ST} values between sites for pearl millet

		L2	L3	L4	M1	M2	M3	M4	M5	M6
**Lowlands**	**L1**	0.007	0.020 *	0.003	0.005	0.005	0.009	0.001	0.010	0.011
	**L2**		0.012	0.000	0.013	0.016	0.014	0.000	0.014	0.008
	**L3**			0.006	0.011	0.013	0.001	0.010	0.014	0.001
	**L4**				0.015	0.003	0.008	0.003	0.015	0.004
**Midlands**	**M1**					0.009	0.000	0.000	0.007	0.009
	**M2**						0.003	0.001	0.015	0.006
	**M3**							0.002	0.006	0.009
	**M4**								0.014	0.007
	**M5**									0.015

***: P < 0.05**

Figure S1. ***Distribution of the major pearl millet*** *(N = 238 variety-farm)* ***and sorghum*** *(N = 367 variety-farm)* ***named varieties among sites****. Barplots display the relative abundance of the different named varieties in each site.* Top: Pearl millet, Bottom: Sorghum

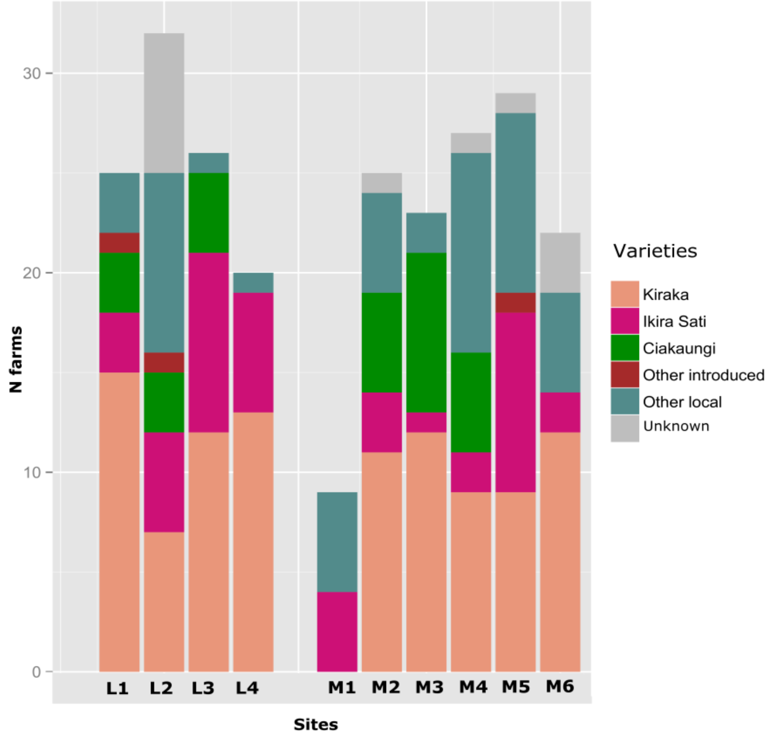

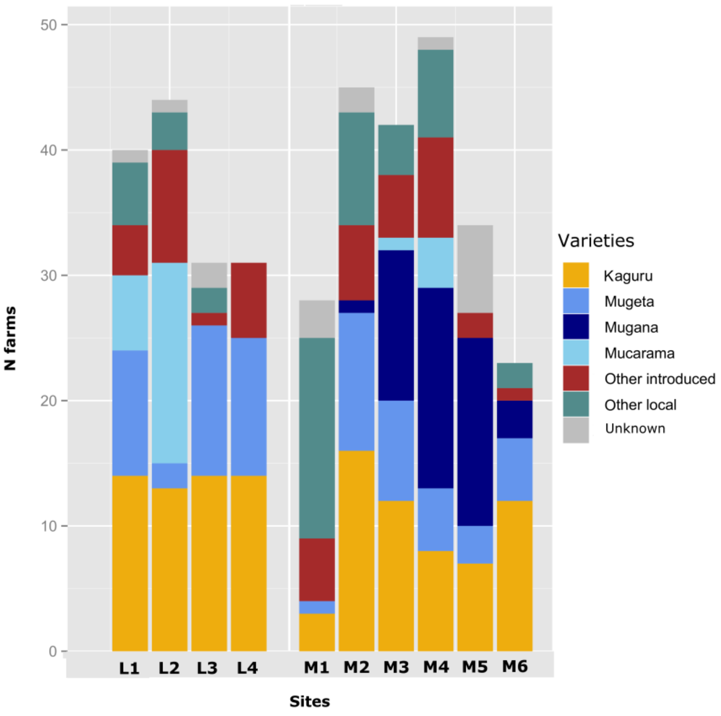

Figure S2. Results of STRUCTURE assignment for pearl millet (A., $N = 286$, $K = 2$)**, and sorghum** (B., $N = 321$, $K = 5$)**.** Individual genotypes assignment to the each of the K populations (each individual is displayed by a vertical bar segmented in K colored sections, representing the proportion of its genome assigned to each population).

A.

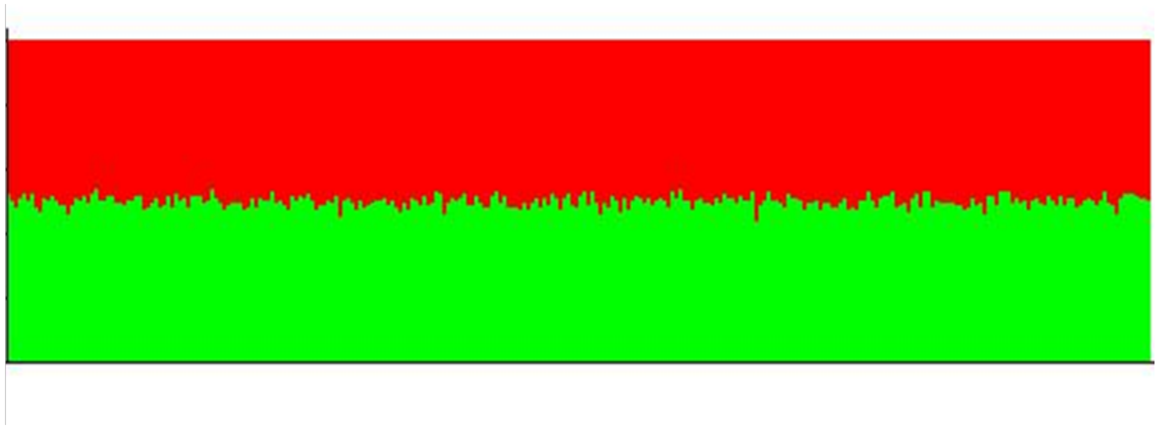

B.

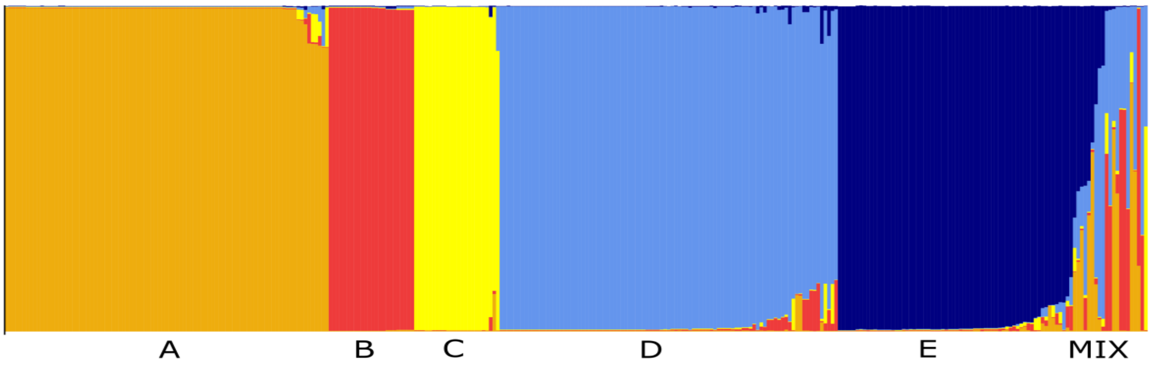

Figure S3. DAPC scatterplot (DA 1 and 2) **of the five African genetic groups inferred using STRUCTURE** (*N* = 1799). G1: durra, bicolor and intermediates from Northeastern Africa; G2: kafir and intermediates from South Africa; G3: caudatum and intermediates from Central and East Africa; G4: guinea and intermediates from West Africa; G5 caudatum, guinea and intermediates from Southeastern and Central Africa.

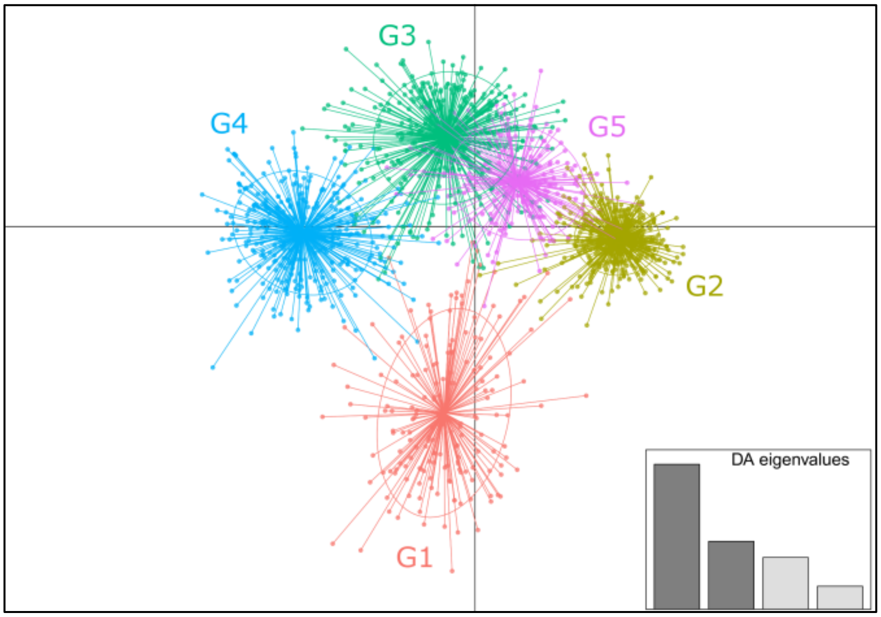

Supplement: Supplementary file 1 — Table S1. Genetic sampling summary. Table S2. Number of samples collected for each sorghum and pearl millet named variety and number of farms where they were collected (between brackets) for each site. Table S3. Summary of information and genetic diversity estimates per locus. Table S4. Pairwise FST values between sites for pearl millet Figure S1. Distribution of the major pearl millet (N = 238 variety‐farm) and sorghum (N = 367 variety‐farm) named varieties among sites. Figure S2. Results of STRUCTURE assignment for pearl millet (A., N = 286, K = 2), and sorghum (B., N = 321, K =5). Figure S3. DAPC scatterplot (DA 1 and 2) of the five African genetic groups inferred using STRUCTURE (N = 1799). [file EVA-9-1241-s001.pdf]
